# Interplay of Meissner Currents and Magnetic Vortices in NbSe_2_ Probed by Tunneling Spectroscopy

**DOI:** 10.1021/acs.nanolett.6c02651

**Published:** 2026-09-07

**Authors:** Maya Klang, Shahar Simon, Oded Millo, Hadar Steinberg

**Affiliations:** † The Racah Institute of Physics, 27219 The Hebrew University of Jerusalem, Jerusalem 91904, Israel; ‡ The Center for Nanoscience and Nanotechnology, 26742 Hebrew University, Jerusalem 91904, Israel

**Keywords:** superconductivity, 2D materials, transition metal dichalcogenides, tunneling spectroscopy, magnetic vortices

## Abstract

Mapping edge supercurrents in thin superconducting films and resolving the Meissner and vortex contributions provide important insights into the nature of type II two-dimensional superconductors. Here, we perform tunneling spectroscopy measurements on thin exfoliated NbSe_2_ flakes to probe Doppler shifts of the quasiparticle density of states and extract the current density at the flake edge with a resolution of 5–10 *μ*A/*μ*m. We observe a distinct hysteretic response of the tunneling spectra under small out-of-plane magnetic fields, allowing a deconvolution of the Meissner and vortex current contributions. The isolated contributions of Meissner currents to the Doppler shift are corroborated using a numerical solution of the London equations. Our results demonstrate that tunneling devices are highly effective for monitoring non-equilibrium and dynamical effects in thin superconducting films.

## Graphical abstract

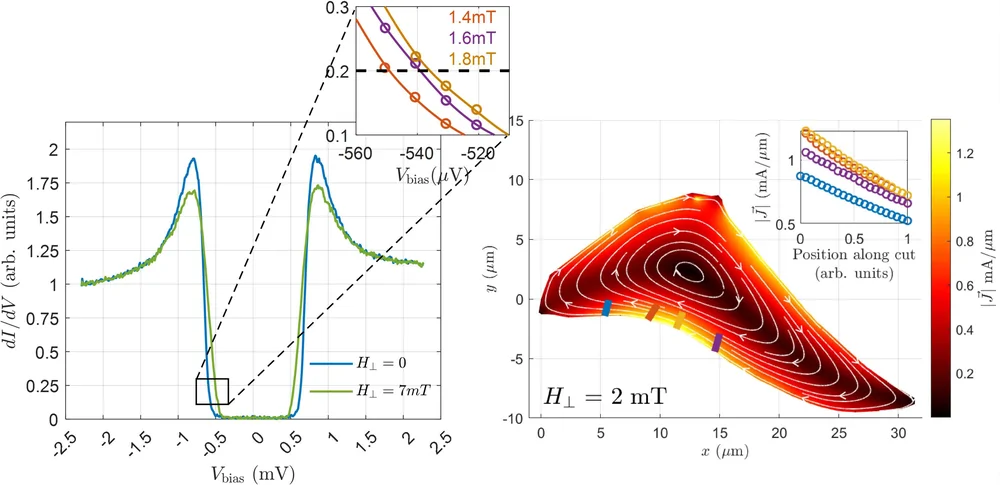

Mapping the current distribution in a superconductor has been a long-standing challenge. Several different probes, measuring magnetic field and deconvolving the current, have been used to this end: scanning Hall probe measurements,^1^ scanning superconducting quantum interference devices (sSQUID),^2–7^ nitrogen vacancy magnetometry,^8,9^ and magneto-optical methods.^10^ Instead of measuring the current-induced magnetic field, it is possible to probe the local supercurrent through its effect on local superconducting properties, for example, using tunneling spectroscopy. Scanning tunneling microscopy and spectroscopy (STM and STS)^11–13^ were used to measure the Doppler shift,^14^ i.e., the shift in quasiparticle (QP) energies, according to

1 $$E_{k}={\left({\varepsilon_{k}}^{2}+\Delta^{2}\right)}^{1/2}+mv_{F}\cdot v_{s}$$

where *E*_k_ is the momentum-dependent quasiparticle energy, *ε*_k_ is the normal-state energy measured relative to the chemical potential, *Δ* is the superconducting gap, *m* is the effective mass, **v**_F_ is the Fermi velocity, and **v**_s_ is the condensate drift velocity. This relation holds as long as the order parameter is not significantly modified by the magnetic field.

Currents in a superconductor may also be probed by engaging a fixed tunneling electrode, which is either metallic or superconducting. Such junctions are compatible with operation at millikelvin-range temperatures in a dilution refrigerator, are mechanically stable, and offer excellent energy sensitivity, while sacrificing real-space resolution. Anthore et al.^15^ used copper electrodes to tunnel into an evaporated Al wire to demonstrate the equivalence of the effects of bias current and applied out-of-plane magnetic field on the tunneling spectrum. Their results were in good agreement with the Usadel formalism for a dirty-limit superconductor,^16,17^ with the quasi-1D geometry ensuring a homogeneously penetrating magnetic field.

Here, we use tunnel junctions in which the superconducting material is thin exfoliated 2H-NbSe_2_ and the tunnel barrier is exfoliated 2H-WS_2_ (henceforth simply NbSe_2_ and WS_2_, respectively), stacked using standard van der Waals (vdW) fabrication techniques (see section 1 of the Supporting Information for details). We tunnel into the NbSe_2_ flake using several closely spaced junctions along its edge and measure differential tunneling conductance d*I*/d*V*, proportional to the density of states (DOS) at low temperatures. We position our junctions along locally straight edges of the flake and far from step edges, thus minimizing the effect of vortex pinning due to roughness^18^ in the vicinity of our electrodes. These choices of material and measurement scheme expand on the results of Anthore et al. Previous tunneling studies of NbSe_2_ in magnetic field have characterized the response of bulk samples,^19^ which is dominated by depairing in the diffusive band associated with the Se atoms. Our samples are much thinner, in the regime where the Se Fermi surface is absent.^20^ Thus, the system responds as a clean superconductor in which the QP lifetime effects are negligible and the main consequence of the magnetic field on the Nb-derived spectral features^21^ is the Doppler shift rather than depairing. Additionally, the 2D geometry allows for spatial variations in the penetrating field and current density.

We find that the measured tunneling spectra are sensitive to Doppler shifts as small as a few microvolts, corresponding to a sensitivity of a few microamperes per micrometer in current density. The spectra are measured on small (∼20 *μ*m) and thin (<10 nm) superconducting samples, where the geometric dimensions cause a considerable spatial overlap of Meissner currents and vortex-circulating currents, a unique property of mesoscopic samples absent in the bulk. The Doppler response displays a hysteretic behavior with respect to magnetic field, allowing a full separation of the Meissner current and the vortex current contributions.

Figure 1 shows a typical tunnel device structure and basic measurement data. Panel a depicts an optical micrograph of a representative tunneling heterostructure, with its schematic side view presented in panel b. In panel c, we display a tunneling spectrum of a thin, 15-layer NbSe_2_ sample (junction 1, thickness estimated by optical contrast, ∼10 nm) in zero magnetic field and base temperature (blue). The spectrum exhibits the key features typical of a high-quality tunnel junction:^21,22^ a region of strongly suppressed tunneling conductance (“hard gap”) around the Fermi energy and QP peaks increasing to a conductance of approximately twice the normal conductance (*G*_N_) found at an increased bias. We observe a superconducting gap of ≈0.8 mV, narrower than the gap typically reported for bulk samples, around 1.2 mV, in accordance with the thickness-modified trend shown by Kuzmanović et al.^20^ and Khestanova et al.^23^ The effect of small out-of-plane magnetic field *H*_⊥_ is the focus of this work. The spectrum measured in *H*_⊥_ = 7 mT (green) is overlaid on the spectrum measured in *H*_⊥_ = 0. The small field suppresses the QP peaks and simultaneously reduces the region of suppressed conductance (the “spectral gap”^14^).

**Figure 1:**
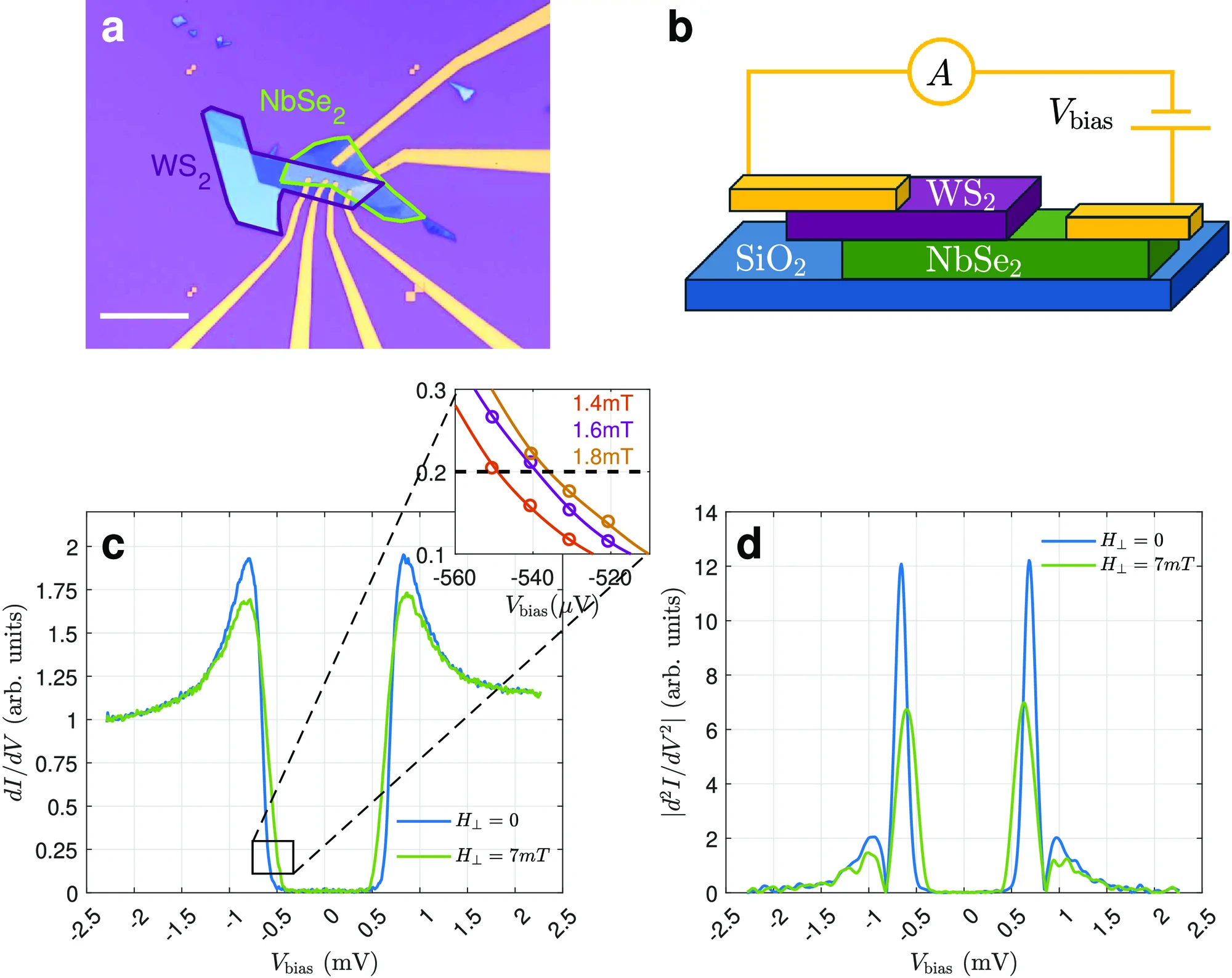
Device design and tunneling spectrum. (a) Optical micrograph of a representative tunnel device heterostructure on a SiO_2_ substrate: green outline, NbSe_2_; purple outline, WS_2_ serving as the tunnel barrier. Scale bar, 20 μm. (b) Schematic of a single tunnel junction, with colors corresponding to panel a. Bias voltage *V*_bias_ is applied between the source and drain electrodes on the barrier above the NbSe_2_ and directly on the NbSe_2_, respectively; tunneling current *I* and tunneling spectrum d*I*/d*V* are recorded simultaneously. (c) Representative tunneling spectrum of a 15-layer thick NbSe_2_ sample in 0 (blue) and 7 mT (green) out-of-plane magnetic field. The inset shows the gap exit region in three consecutive magnetic field values. The threshold value for *V*_exit_ is marked by the dashed black line. Measured values marked as circles, overlaid with a piecewise third-order polynomial smoothing spline for the sake of clarity. (d) Numerical derivatives (in absolute value), |d^2^ *I*/d*V* ^2^| , of the spectra in panel c.

To systematically characterize the spectral gap, we define the exit voltage *V*_exit_ as the voltage at which the spectrum reaches 20% of *G*_N_, marking the “exit” from the gap. The part of the conductance curve crossing the 20% threshold is highlighted in the inset of panel c for three additional field values. The softened rise of the DOS at the gap edge and the narrowing of the spectral gap in increasing field are more apparent in the derivative of the spectrum, |d^2^ *I*/d*V* ^2^|, shown in panel d at *H*_⊥_ = 0 (blue) and *H*_⊥_ = 7 mT (green). Note that the peaks correspond to the highest slopes in conductance and evolve in field similarly to *V*_exit_. To account for the effect of the non-uniformity of the current under the electrode, we decompose the spectra into a distribution of Doppler-shifted DOS using a Tikhonov regularization algorithm^24,25^ as an approximation of the spatial averaging of the measurement. The details are given in section 4 of the Supporting Information and Figures S2 and S3. We find that *V*_exit_ generally remains a good proxy for the shifting of the gap distribution with magnetic field (with the exception of junction 3 (see below)).

In Figure 2, we present the evolution of |d^2^ *I*/d*V* ^2^| under increasing *H*_⊥_ (panel a, 0 to 7 mT) and decreasing *H*_⊥_ (panel b, 7 to 0 mT). The two measurements show qualitatively different behavior in response to the magnetic field. To quantify this difference in behavior, *V*_exit_ for junction 1 is plotted in blue as a function of the magnetic field in panel c. The behavior of *V*_exit_ can be broken down into four regimes, as shown in panel d. In increasing field, *V*_exit_ first decreases linearly up to *H*_⊥_ ≈ 2.5 mT (regime I) and then saturates (regime II). In decreasing field, in regime III, *V*_exit_ increases linearly at a steeper slope (compared to that of regime I), recovers its zero-field value at ≈5 mT, and remains stable when the field is decreased further to zero (regime IV). This general behavior is punctuated by occasional discrete jumps in increasing field, where *V*_exit_ jumps toward higher values and then gradually returns to its previous behavior, for example, at ≈4.4 mT for junction 1. We point out that this hysteretic behavior depends solely on the magnitude of the field, irrespective of its sign: ramping away from zero, in the positive and negative directions, elicits the same response of linear spectral gap reduction and saturation. Similarly, ramping back toward zero restores the zero-field state at ±5 mT. For the sake of simplicity, ramping the field away from zero will be termed “increasing”, and ramping towards zero as “decreasing”. Similar behavior is observed when the magnetic field range is wider.

**Figure 2:**
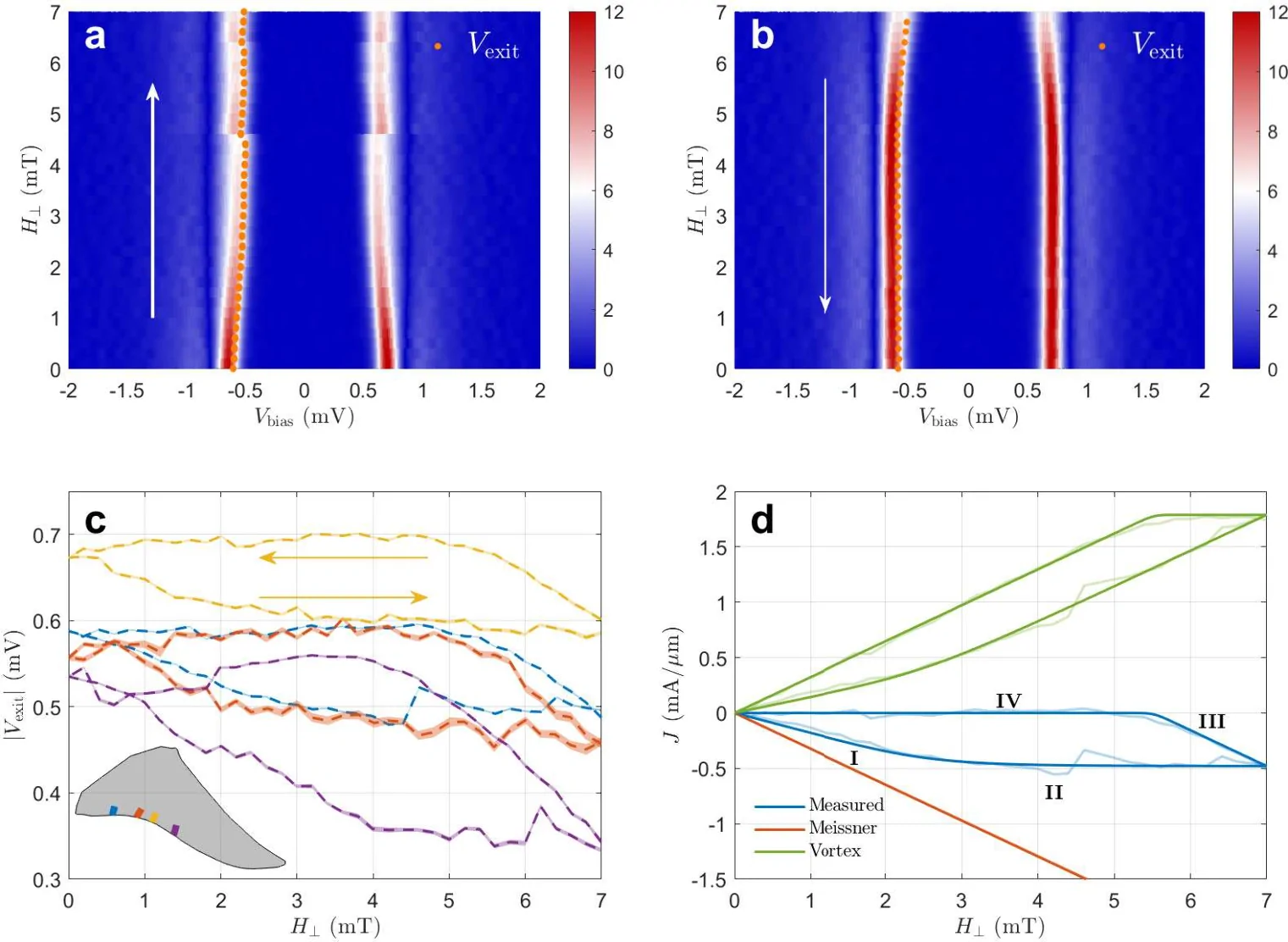
Hysteretic behavior in magnetic field. (a) Response of the spectrum, |d^2^ *I*/d*V* ^2^|, to increasing out-of-plane magnetic field for junction 1 (blue in the schematic in panel c). *V*_exit_ is highlighted with orange dots. (b) Data similar to those presented in panel a, in decreasing field. The data sets in panels a and b were recently published by Simon et al.,^26^ but for a different type of analysis. (c) *V*_exit_ values extracted from panels a and b (blue), displaying a linear decrease and saturation in increasing field, and then a rapid linear restoration of the zero-field value in decreasing field. Similar data from additional junctions are colored red, yellow, and purple. A schematic of the NbSe_2_ flake is shown in gray, with electrode positions colored corresponding to their measured data. The width of the shaded regions along the curves indicates the uncertainty in *V*_exit_ (see below for details). The field scan direction is indicated by arrows for the yellow data set. (d) Schematic evolution of the current under an electrode as a function of *H*_⊥_. The contribution from Meissner currents (red) is linear and reversible. The total current (blue) decreases linearly (regime I) and saturates (regime II) in increasing field and then increases more steeply (regime III) with the slope of the Meissner contribution when the field is reduced again until it saturates back to zero (regime IV). The contribution from vortices (green) is the difference between the total current and the Meissner contribution. Data derived from junction 1 using eq 2 are plotted in semitransparent lines for reference.

We note that our spectra exhibit a hard gap throughout the measurement even when vortices are present in the sample. This is in contrast to previous studies of the mixed state in NbSe_2_, where a noticeable change in the DOS was observed at zero bias.^19^ The indifference of the subgap spectrum to the presence of vortices arises from properties of our device architecture and the weak vortex pinning potential in NbSe_2_.^27^ Although vortices enter at the edge of the sample, in a semisteady state and at a low field they tend to drift toward the center of the flake,^18,28,29^ far from the junction. Since the tunneling spectrum is measured semilocally, averaging over the area of the junction, it is insensitive to the DOS contributed by Caroli–de Gennes–Matricon states^30^ bound to the vortex cores.

The behavior of *V*_exit_ is replicated across all of our measured devices and at various flake thicknesses. Three additional data sets taken on adjacent electrodes on the 15-layer flake (junctions 2–4) are shown in Figure 2c in red, yellow, and purple. These show a slight variation in the initial value of *V*_exit_, as expected from typical fluctuations in thickness.^23,31^ However, the slope of *V*_exit_ versus *H*_⊥_ is similar across all four junctions, with variations discussed in depth below. Figure 2d summarizes schematically this generic behavior, sketched in blue: linear decrease in *V*_exit_ (regime I), saturation (regime II), linear increase upon reversal of the field (regime III), and recovery of the zero-field value (regime IV). Additional data of a seven-layer device are presented in Figure S1.

We propose that the effect we observe is the consequence of the interplay between the two main modes of magnetic field response in a small-area type II superconducting sample: the formation of Meissner screening currents and the penetration of flux as vortices. These effects work in conjunction with the semilocal nature of the tunneling measurement, where the sample is probed within ∼1 μm of its edge. The positioning of tunnel junctions along the edge of the flake ensures that they are significantly affected by Meissner currents, which increase toward the edge of the sample.

Thin NbSe_2_ is a high-*κ* ^32^ type II superconductor. In such a material in the thin limit, *H*_c1_ is strongly dependent on geometry, and in fact, the transition is often too low to be observed due to edge effects. This is due to nucleating vortex entry resulting from a local suppression of the order parameter, a local lowering of the Bean–Livingston barrier via a change in the Meissner current density,^18,28,33^ or both. Consequently, when a field is applied, the total current is comprised of two contributions. One is from Meissner screening currents that expel the applied field. The other is from currents that circulate around the vortex cores, counterpropagating with respect to the Meissner currents.^12^ In our samples, thickness *d* is much smaller than the London penetration length *λ*, and consequently, we observe Pearl-type vortices^34^ whose currents decay over a distance *Λ* = 2*λ* ^2^/*d* ∼ 10 *μ*m and are thus appreciable throughout the sample.

The simplest form of accounting for the effect of supercurrent on the spectrum of a clean superconductor is through the Doppler shift of QP energies, caused by the contribution of a magnetic vector potential, including both Meissner and vortex contributions, to the QP energy. This results in a shifted QP spectrum as shown in eq 1. In terms of current, the shift of the lowest QP energy takes the form of

2 $$\Delta E=-mv_{F}v_{s}=-\frac{m}{en_{s}}v_{F}\left|J_{s}\right|$$

where **J**_s_ is the 2D supercurrent density and *n*_s_ is the superconducting carrier density (see section 3 of the Supporting Information for details). Here we use the carrier density in a single NbSe_2_ layer^35^ and multiply by the number of layers. The narrowing of the spectral gap can then be tracked by the evolution of *V*_exit_; hence, *V*_exit_ serves as a probe for the area-averaged total current under the electrode.

Figure 2 shows that *V*_exit_ evolves linearly in regime I. Naively, this linear trend can be interpreted as solely due to the Meissner effect. Below, we show that, in fact, vortices contribute counterpropagating current even at these low fields. In regime II (above *H*_⊥_ = 2.5 mT in the blue, red, and yellow data), *V*_exit_ saturates and remains almost constant up to the maximum applied field (*H*_⊥_ = 7 mT). This saturation indicates that the added vortex and Meissner currents fully compensate for each other. This increased effect of the vortex currents is caused either by enhanced vortex penetration due to the reduction of the Bean–Livingston barrier^36^ with field or by the fact that mutual vortex repulsion favors positioning new vortices closer to the edge.

In regime III, when the field is decreased back to zero (panel b), the spectral response does not retrace the evolution seen in panel a. Instead, the gap rapidly increases, and the spectrum recovers its zero-field state, evident in the loop-shaped behavior of *V*_exit_ (Figure 2 c). For junction 1 (panel b and blue curve in panel c), this occurs at *H*_⊥_ ≈ 5 mT. This rapid increase in *V*_exit_ corresponds to the decrease in Meissner currents, while the vortices remain pinned inside the sample, following the Bean–Livingston model. Careful observation of Figure 2c reveals that the slopes of *V*_exit_ versus *H*_⊥_ differ upon increasing and decreasing the field, the latter being steeper by ∼75%. This is explained by the fact that vortex penetration while increasing the field adds a counterpropagating current contribution, slowing the increase in the total edge current. Consequently, we argue that the steeper slope in decreasing field is the true Meissner response of the sample, unimpeded by the effect of vortices.

At this point in the measurement, the vortex and Meissner counterpropagating currents reach the minimum-kinetic energy state. This is manifested as a return to the zero-field spectrum with a maximal gap, consistent with the full cancellation of the two contributions within the sensitivity of the measurement. In regime IV, decreasing the field further from 5 mT allows the vortices to exit the sample and Meissner currents to vanish until the field crosses to the negative side.

Figure 2d is a generic schematic showing our suggested decomposition of the total current density measured under a tunneling electrode (blue) to its Meissner (red) and vortex (green) contributions. As discussed above, regime III shows the linear trend of the genuine Meissner currents, where the vortices are pinned. Thus, the Meissner contribution can be deduced from it as the red trend line, parallel to the blue trace in regime III and passing through the origin. As the total current is the sum of two contributions, the vortex contribution, colored green, can be found by subtracting the Meissner current contribution from the total current density. We demonstrate this procedure in Figure 2d for the data measured on junction 1, plotted in blue in panel c, and display it in semitransparent lines alongside the schematic in matching colors.

To corroborate our proposed mechanism, we also performed a field cooling measurement, as shown in Figure 3. We start our measurement at zero field and increase it to *H*_⊥_ = 5 mT, where the spectral gap is visibly narrowed. With the field still applied, we heat the sample to above *T*_c_ = 7.2 K, allowing flux to freely penetrate the sample. Then, we cool the sample back to the base temperature of ≈25 mK. Because of the preexistence of flux in the sample, vortices are preformed in the absence of an energy barrier, and the sample reaches a minimum-energy current distribution. In particular, semilocal currents under the electrode are below the measurement sensitivity, and a zero-field DOS, with no Doppler shift, is measured. We then continue measuring spectra in increasing fields from 5 to 10 mT, and as expected, we see the process qualitatively repeating itself, albeit with a key difference. Due to the higher field, the Bean–Livingston barrier is lower, and as a result, the slope of *V*_exit_ is noticeably reduced, from 35 to 28 *μ*V/mT, indicating enhanced vortex penetration.

**Figure 3:**
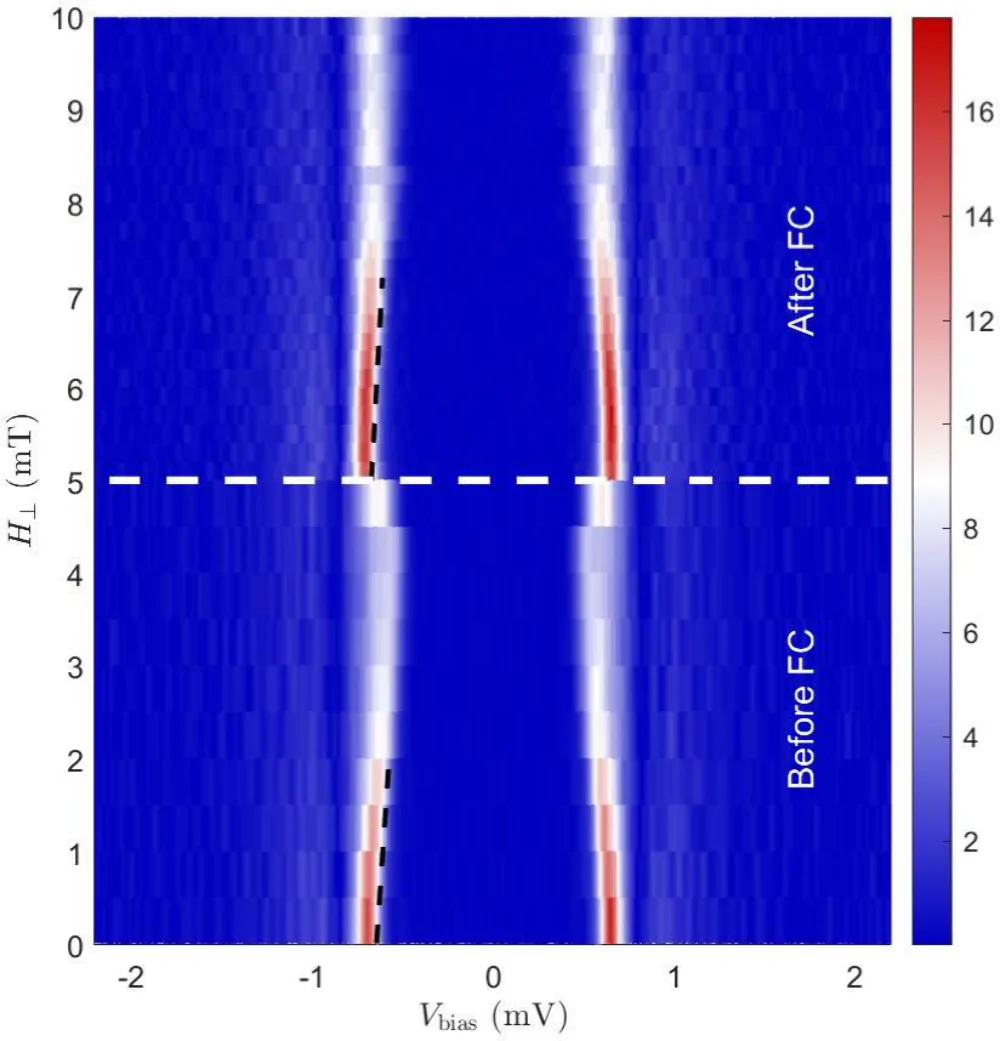
Spectra measured with field cooling. A measurement similar to that shown in Figure 2a up to 5 mT. At 5 mT, the sample was warmed above *T*_c_ and then cooled to the base temperature again. Then the measurement was resumed up to 10 mT. It is apparent that the zero-field spectrum was measured after the field cooling despite the finite applied field. Black dashed lines indicate the slopes mentioned in the text.

Modeling the effect of low out-of-plane fields on the width of the spectral gap further supports our explanation. This is accomplished by numerically solving the London equations for the Meissner currents in the flake and calculating the resulting Doppler shift. Figure 4a shows the calculated Meissner current density map in the flake **J**_s_(**r**) at *H*_⊥_ = 2 mT, accounting for the geometry, thickness, and effective penetration depth^3,34,37^ of the flake (calculation performed using SuperScreen^38^). The effect of the resulting current map on *V*_exit_ for each electrode is then calculated according to eq 2 for all fields, using an *n*_s_ of 1.9 × 10^19^ m^–2^, measured by Xi et al.^35^ and fitting an effective *v*_F_ of 1.05 × 10^5^ m/s, within the range of values measured on the Nb-derived Fermi surfaces by Kiss et al.^39^ and Rahn et al.^40^ on bulk samples. In Figure 4b, the resulting linear trends for junctions 1–4 are superimposed on the measured data in regime III (see Figure 2c), where *V*_exit_ is affected by only the Meissner current. Good agreement is found for junctions 1, 2, and 4 when using the same *n*_s_ and *v*_F_ given above, and also for the seven-layer thick sample shown in section 2 of the Supporting Information. The discrepancy of the model prediction with the data from junction 3 can be explained by current redistribution under the electrode, as shown in Figure S3.

**Figure 4:**
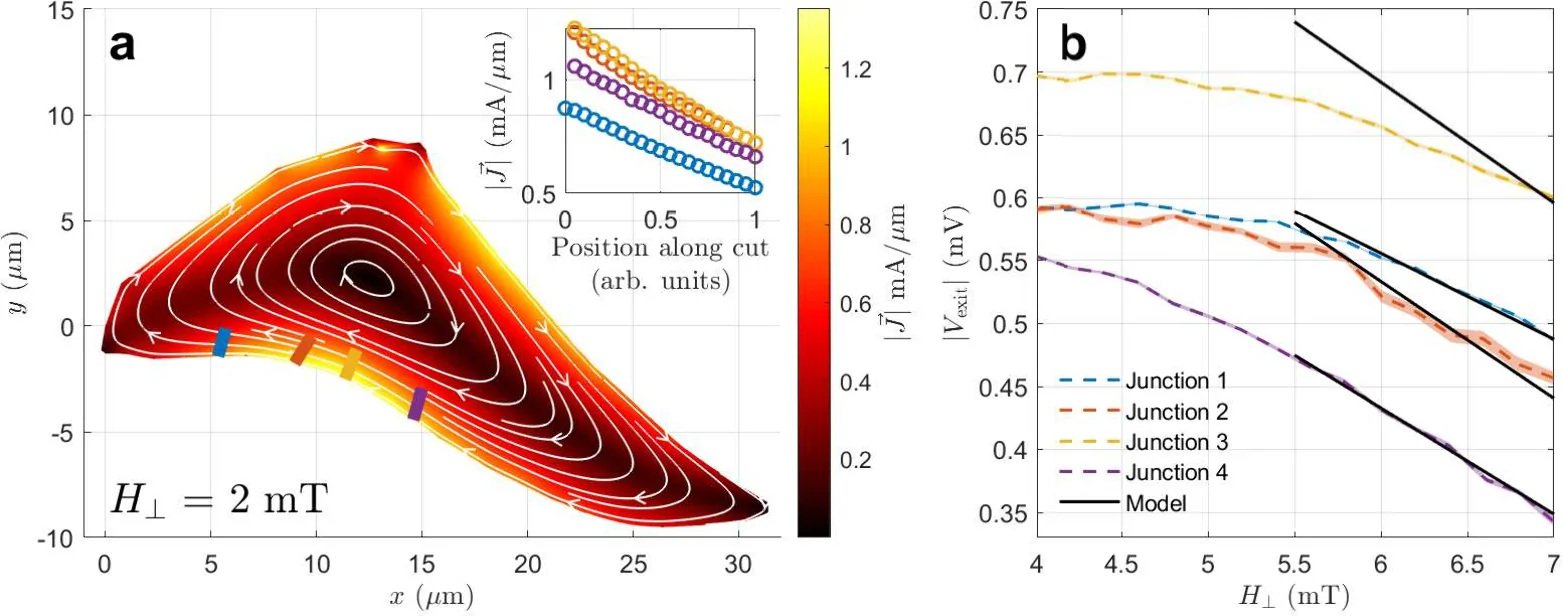
Current density calculation and comparison with the measurement. (a) Representative current density map, calculated using SuperScreen^38^ at *H*_⊥_ = 2 mT. The inset shows the current density profiles taken along the approximate positions of the electrodes, with colors corresponding to the junctions in panel a. Horizontal axis normalized by the length of each junction, ≈1 *μ*m. (b) *V*_exit_ measured on all four junctions in regime III and the values calculated using eq 2. The shaded region along each curve corresponds to the uncertainty in *V*_exit_; model predictions are shown as black lines.

Discrete jumps in *V*_exit_ seen in the data are also explained by this model. Figures S4 and S5 display current density maps in the presence of a single vortex, at zero field and at finite field, respectively. They both indicate that such jumps can be accounted for by single-vortex penetration close to the electrode, as the model reproduces strikingly similar jumps. This analysis also suggests large jumps such as the prominent feature at *H*_⊥_ = 4.4 mT in Figure 2a are the result of a large number of vortices penetrating at once, even if they penetrate quite far from a junction, as we demonstrate by calculating the response of the current density to the abrupt penetration of nine vortices, several micrometers from the electrodes (Figure S6). The penetration of a larger number of vortices, or a similar number at a position closer to the electrode, may plausibly account for the larger jumps in the data. Effects of discrete vortex penetration events are seen in Josephson junctions connecting 2D superconducting leads,^41,42^ where the vortex redistributes the edge currents, resulting in jumps in the critical current.

Our results demonstrate the efficacy of tunnel junctions as probes for local currents through their high sensitivity to energy shifts. Based on eq 2, we can estimate the uncertainty of the local current density as $\Delta J=\frac{e^{2}n_{s}}{{\mathrm{mv}}_{F}}\Delta V_{\mathrm{exit}}$ , with the uncertainty in *V*_exit_ given by the equation *ΔV*_exit_ = (∂*G*/∂*V*)^−1^|_*V*_exit__ *ΔG*. Using the standard deviation of the conductance measured inside the hard gap to estimate *ΔG*, we find *ΔV*_exit_ ∼ 1–2 μV. Although the exit from the gap is rounded by *k*_B_ *T*/*e* ≈ 30 *μ*V, a rigid shift of this feature, such as the one caused by a Doppler shift, can be measured at a far better resolution. This is seen in the inset of Figure 1c, showing spectra measured at fields that differ by 0.2 mT having consistent rigid shifts of a few microelectronvolts, much smaller than *k*_B_ *T*/*e*, in the gap exit region. The uncertainty in *ΔV*_exit_ noted above corresponds to *ΔJ* = 5–10 *μ*A/*μ*m for the average current under the junction. As shown in the inset of Figure 4a and discussed in section 4 of the Supporting Information, the current density varies along the longitudinal position in the junction, extending ∼1 *μ*m into the flake, by about 30%, averaging the *V*_exit_ variation over a range of different Doppler shifts. Consequently, smaller junctions are expected to improve the precision of this technique. We conclude that semilocal tunnel junctions are sensitive to Meissner currents and to the effects of vortices both near and far from the junction position and enable the separation of the two contributions. This analysis can be applied to any superconducting system where the screening length is comparable to the size of the sample. This can be easily achieved in exfoliated flakes of vdW materials but is by no means limited to them. Any clean-limit superconductor where the ratio *n*_s_/*mv*_F_ is sufficiently small to generate a measurable Doppler shift will do. It also lends itself to measuring Doppler shift effects in current-carrying samples.

## Supplementary Material



## Notes

Extended experimental methods, extended data on an additional seven-layer thick device, a derivation of the dependence of the Doppler shift on current density, a detailed protocol for the decomposition of spectra, and additional numerical results (PDF)
