# Supplementary material for: Interplay of Meissner Currents and Magnetic Vortices in NbSe2 Probed by Tunneling Spectroscopy
Source: Nano Lett. 2026 Sep 7;26(37):12309–15. doi: 10.1021/acs.nanolett.6c02651 (PMC13614297; doi:10.1021/acs.nanolett.6c02651)
Supplement: Supplementary file 1 [file nl6c02651_si_001.pdf]

Supporting Information: The Interplay of Meissner Currents and Magnetic Vortices in $NbSe_2$ Probed by Tunneling Spectroscopy

Maya Klang[1,2,*], Shahar Simon[1,2,*], Oded Millo[1,2,**], and Hadar Steinberg[1,2,**]

[1] *The Racah Institute of Physics, The Hebrew University of Jerusalem, Jerusalem 91904, Israel*

[2] *The Center for Nanoscience and Nanotechnology, Hebrew University, Jerusalem 91904, Israel*

[*] *These authors contributed equally to this work.*

[**] *hadar@phys.huji.ac.il; milode@mail.huji.ac.il*

August 28, 2026

Contents

1 Experimental methods **1**

2 Data measured on a 7-layer device **2**

3 Derivation of the dependence of V_{exit} on field **2**

4 Decomposing spectra into distributions **3**

5 Current maps of single vortices at zero field **6**

6 The effect of a single vortex at finite field **7**

7 The effect of multiple vortices at finite field **7**

1 Experimental methods

We fabricate our tunnel junctions using the standard polycarbonate (PC) pickup techniques that are extensively used in TMD heterostructure device fabrication. We exfoliate our materials onto p-doped Si wafers having a 285 nm thick thermally-grown SiO_2 layer for ideal optical contrast, which is crucial for preliminary optical determination of flake thickness. We then pick up the WS_2 flake to be used as a tunnel barrier, which in our experience performs best at a thickness of 3-4 layers, by heating our PC film to a temperature of 70°C and disengaging slowly. We then lower the barrier flake on top of the designated $NbSe_2$ flake, making sure it overlaps the area of interest in the superconductor, heat up to 90°C and disengage to pick up the $NbSe_2$ flake. Next, we transfer the two-flake stack onto a clean Si-SiO_2

chip and heat up to 160°C to fully melt the PC film. Finally, we dissolve the melted PC by immersing the chip in chloroform for 5 minutes, ending up with a clean chip with the heterostructure on top. All exfoliation and flake transfer procedures are performed in an Ar atmosphere inside a glove-box with typical water and oxygen levels well below 10 ppm. We subsequently perform standard electron-beam lithography to pattern tunneling and Ohmic-contact electrodes, typically Au/Ti of thicknesses 80 nm/8 nm. To ensure good Ohmic contacts in our drain electrodes, we evaporate these electrodes in a separate session, where we perform a short milling step in Ar plasma inside the evaporator to remove oxides and organic residue from the surface of the $NbSe_2$ flake, immediately before evaporation.

Measurements are performed in a Bluefors LD250 cryostat with a base temperature of ≈25 mK, using heavily filtered DC wiring. Typically, our tunnel junctions exhibit normal resistances (at base temperatures) on the order of 10kΩ, ensuring a tunneling-dominated current while still having low enough noise levels for clean measurements, characterized by zero-bias conductance that is typically two orders of magnitude lower than the normal conductance. Conductance measurements are carried out using a Zurich Instruments MFLI lock-in amplifier, with AC excitations on the order of 10μV, providing high energy resolution. Out-of-plane magnetic fields of up to 10mT were applied, and care was taken to preserve the magnetic history, always completing a full magnetic field loop.

2 Data measured on a 7-layer device

Data measured at two junctions on a 7-layer thick flake of $NbSe_2$ are shown in Fig. S1. We note that in order to reduce noise in the data, we took V_{exit} to be the voltage at which the conductance rises above 30% of the normal value, as opposed to 20% in the main text. Notably, the shift to the QP spectrum is much weaker compared to that observed for the 15-layer flake, in keeping with the thinner sample having a longer Pearl length and its screening becoming very partial. Linear slopes extracted from the model in panel d are fitted using the same value for v_F as for the 15-layer flake data in the main text.

3 Derivation of the dependence of V_{exit} on field

The following derivation is based on Ref.[3]. In the Ginzburg-Landau formalism for superconductors, the free energy density of a superconductor in the presence of a magnetic field is, to fourth order

$$f = f_n + \alpha|\psi|^2 + \frac{\beta}{2}|\psi|^4 + \frac{1}{2m}\left|\left(\frac{\hbar}{i}\nabla - e\mathbf{A}\right)\psi\right|^2 + \frac{B^2}{2\mu_0}. \tag{1}$$

Focusing on the term arising from fields and gradients and writing $\psi = |\psi|e^{i\varphi}$, it takes the form

$$\frac{1}{2m}\left[\hbar^2\left(\nabla|\psi|\right)^2 + \left(\hbar\nabla\varphi - e\mathbf{A}\right)^2|\psi|^2\right]. \tag{2}$$

The second term is associated with the kinetic energy due to supercurrents. This energy density contribution must also be equal to $\frac{1}{2}mn_s v_s^2$. Using $\mathbf{J} = en_s\mathbf{v}_s$, the identity $|\psi|^2 = n_s$ and the London gauge where $\nabla\varphi = 0$, we find

$$\mathbf{A} = -\frac{m}{e^2 n_s}\mathbf{J}. \tag{3}$$

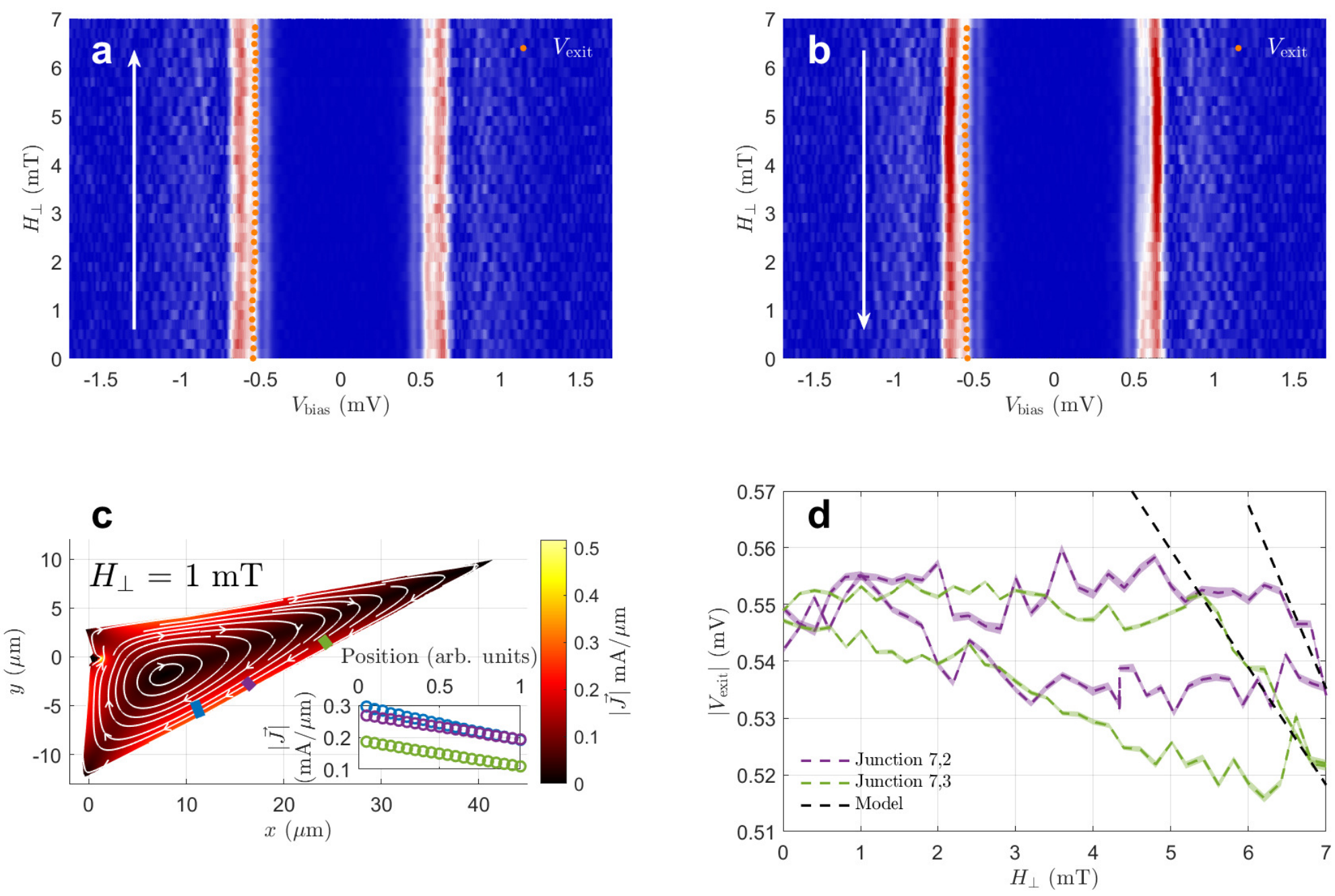

Figure S1: **7-layer data.** (a) Response of the spectrum, $|d^2I/dV^2|$, to increasing out-of-plane magnetic field. V_{exit} is highlighted in orange dots. (b) Similar data to a, in decreasing field. The data sets in panels a and b were recently published by Simon *et al.*,[1] but for a different type of analysis. (c) A representative current density map, calculated using SuperScreen[2] at $H_\perp$ =1 mT. Inset: current density profiles taken along the approximate positions of our electrodes, colors corresponding to the junctions in a. Horizontal axis normalized by the length of each junction, $\approx 1\mu$m. The data set taken on the electrode marked in blue showed a much higher effective temperature than the others and was therefore omitted. (d) Extracted V_{exit} from a and b (purple), displaying a linear decrease and saturation in increasing field, then a rapid linear restoration of the zero-field value in decreasing field. Similar data from Junction 7,3 are shown in green. The width of the shaded regions along the dashed lines indicates the uncertainty in V_{exit} (see main text for details).

This result can then be used to yield

$$|\Delta E| = \frac{e}{m}|\mathbf{p}\cdot\mathbf{A}| = \frac{e}{m}mv_F\frac{m}{e^2n_s}J = \frac{mv_F}{en_s}J. \tag{4}$$

4 Decomposing spectra into distributions

In order to verify the reliability of the procedure used to derive the values of J from our measurements of the spectra and V_{exit}, we perform an additional type of analysis. Spatial variation of J across the area of the tunnel junction is expected to result in a concomitant variation of V_{exit}, such that the actual value we derive for V_{exit} is in fact $\langle V_{\mathrm{exit}}\rangle$, where the averaging is over the area of the junction. As a first approximation, we postulate that the measured spectrum is in fact a spatial average of a distribution of

local spectra $DOS(\vec{r}) = N(\Delta, E, \delta E)$, where δE is the local Doppler shift. At small shifts, we expect the effect of the shift to manifest itself primarily in a change of the order parameter Δ, and the change in the shape of the spectrum to be secondary, such that $N(\Delta, E, \delta E) \approx N(\Delta - \delta E, E, 0)$. For the choice of the model of superconductivity yielding the DOS, we make a compromise between accuracy and ease of computation. As we show in a recent publication by Simon *et al.*,[1] the BCS DOS cannot account for the shallow slope at the exit from the gap, which is exactly the part we are interested in. Conversely, the model that best reproduces thin $NbSe_2$ tunneling spectra is the McMillan two-band model, but it is not straightforward to work the Doppler shift into it due to the presence of several interacting energy scales. We compromise by using the anisotropic gap model proposed by Khestanova *et al.*,[4], where the DOS is given as the average over the Fermi surface of a momentum-dependent BCS DOS

$$N(E, \Gamma, \Delta, \beta) = Re\left[\frac{1}{2\pi}\int_0^{2\pi} \frac{E - i\Gamma}{\sqrt{(E - i\Gamma)^2 - \Delta^2(\theta, \beta)}} d\theta\right], \tag{5}$$

where

$$\Delta(\theta, \beta) = \Delta_0 \left[\beta cos(6\theta) + (1 - \beta)\right]. \tag{6}$$

Here, Γ is the Dynes broadening parameter, $\beta \in [0, 1]$ is an anisotropy parameter, Δ_0 is the maximal gap value and the anisotropy satisfies the symmetry of the underlying crystal.

Extracting the distribution

In order to decompose our spectra, we aim to solve the Fredholm integral equation of the first kind

$$G_{\text{meas}}(V) = \int_{\Delta_{\min}}^{\Delta_{\max}} p(\Delta) N(V, \Delta) d\Delta, \tag{7}$$

for the distribution $p(\Delta)$, where G_{meas} is the measured spectrum, V is the voltage bias, $N(V, \Delta)$ is a parametrized component of the DOS distribution as a function of V and the gap parameter Δ, and $p(\Delta)$ is the distribution of gaps that we solve for. The discretization of this problem results in the matrix equation

$$\mathbf{g}_{\text{meas}} = \mathbf{A}_{\text{kernel}} \mathbf{p}, \tag{8}$$

where $\mathbf{g}_{\text{meas}}$ is the discretized vector that was measured, $\mathbf{p}$ is the discretized distribution and $\mathbf{A}_{\text{kernel}}$ is the kernel matrix constructed by $A_{i,j} = N(V_i, \Delta_j)$. The existence of the physical constraints of the non-negativity and normalization of $p(\Delta)$, and the fact that the columns of $\mathbf{A}_{\text{kernel}}$ are nearly linearly dependent when $d\Delta$ is small, prohibit a simple solution by inversion of $\mathbf{A}_{\text{kernel}}$ even if it was a square matrix. We thus apply these constraints by performing a Tikhonov regularization algorithm.[5,6]

This problem is in essence a constrained, regularized least-squares optimization. We aim to minimize the functional

$$||\mathbf{A}_{\text{kernel}} \mathbf{p} - \mathbf{g}_{\text{meas}}||^2 + \lambda^2 ||\mathbf{L}\mathbf{p}||^2 \tag{9}$$

with respect to $\mathbf{p}$, subject to $p_i \geq 0$ and $\sum_i p_i = 1$, where λ is a positive hyperparameter determining the relative weight of the two terms and $\mathbf{L}$ is a second-derivative matrix. The first term is a data fidelity term, *i.e.*, a simple sum of the squared distance of the measured data from the suggested fit. The second is a smoothing term, which penalizes high curvature of the suggested fit.

It is then trivial that the following equality holds:

$$||\mathbf{A}_{\text{aug}}\mathbf{p} - \mathbf{b}_{\text{aug}}||^2 = ||\mathbf{A}_{\text{kernel}}\mathbf{p} - \mathbf{g}_{\text{meas}}||^2 + \lambda^2||\mathbf{L}\mathbf{p}||^2 \tag{10}$$

if $\mathbf{A}_{\text{aug}} = \left[\begin{smallmatrix}\mathbf{A}_{\text{kernel}} \\ \lambda\mathbf{L}\end{smallmatrix}\right]$ and $\mathbf{b}_{\text{aug}} = \left[\begin{smallmatrix}\mathbf{g}_{\text{meas}} \\ \mathbf{0}\end{smallmatrix}\right]$, with $\mathbf{0}$ a column of zeros with the same number of elements as rows in $\mathbf{L}$. Consequently, the problem is reduced a standard linear least-squares problem which can be fed, *e.g.*, to Matlab's lsqlin function, along with the requirements for a lower bound of zero and unity normalization.

Choice of parameters and the resulting distributions

The choice of the hyperparameter λ requires a simple optimization procedure by calculating the two terms in Eq. 9 for a representative spectrum and taking the λ value that yields the lowest error in both, simultaneously. The range of Δ values was chosen to span the range from $\Delta \approx 0$ up to approximately twice the distance between the QP peaks in the zero-field measured spectrum. For the zero-field DOS, a very small Dynes parameter of 5μeV was chosen to account for the small in-gap conductance, a fitted effective temperature $T = 0.35$ K accounted for thermal broadening and an anisotropy parameter $\beta = 0.06$ yielded the best fit to the data.

Fig. S2 shows the gap distributions for Junction 1 in increasing and decreasing fields in panels a and b, respectively. In all fields, the distribution is described adequately by a Gaussian fit. A line cut in zero field is shown in the inset, with the Gaussian fit overlaid to show the good agreement. A small shoulder in higher gaps is visible in low increasing field and in almost all decreasing fields. This zero-field discrepancy is no surprise, as the anisotropic gap model is widely understood to be an approximation. It is clear that the lobe that starts around 0.78 meV follows a similar path to V_{exit} as displayed by Fig. 2 of the main text. Panel c shows that the width of the Gaussian, σ, normalized by the mean of the distribution $\langle\Delta\rangle$ varies very weakly in field, experiencing only a few percents of broadening, following the requirement that the Meissner current should decrease smoothly to zero in the middle of the flake. This confirms that the distribution of currents in this junction is almost constant across the measurement range and the effect of field is only to increase the current magnitude, shifting the position of the lobe while smearing it very slightly. This is not the case in Junction 3, as explained below. In panel d, we plot the 7 mT spectrum and overlay the gap-distribution reconstruction found by our decomposition algorithm, showing excellent agreement inside the gap.

Discrepancy and current redistribution in Junction 3

In contrast to the stability of the distribution width seen in Junction 1, Junction 3 exhibited notably different behavior. In Fig. S3 we show the gap distribution of Junction 3 in increasing and decreasing fields in panels a and b, respectively. It is remarkably clear that the position of the mean of the lobe hardly changes throughout the measurement. Furthermore, as we show in panel c, the lobe undergoes noticeable broadening. This behavior indicates that the current under the electrode is redistributed by the field while its mean does not change noticeably, in stark contrast to the behavior of Junction 1. This may be due to a current hot spot, forming a current profile that decays faster towards the middle

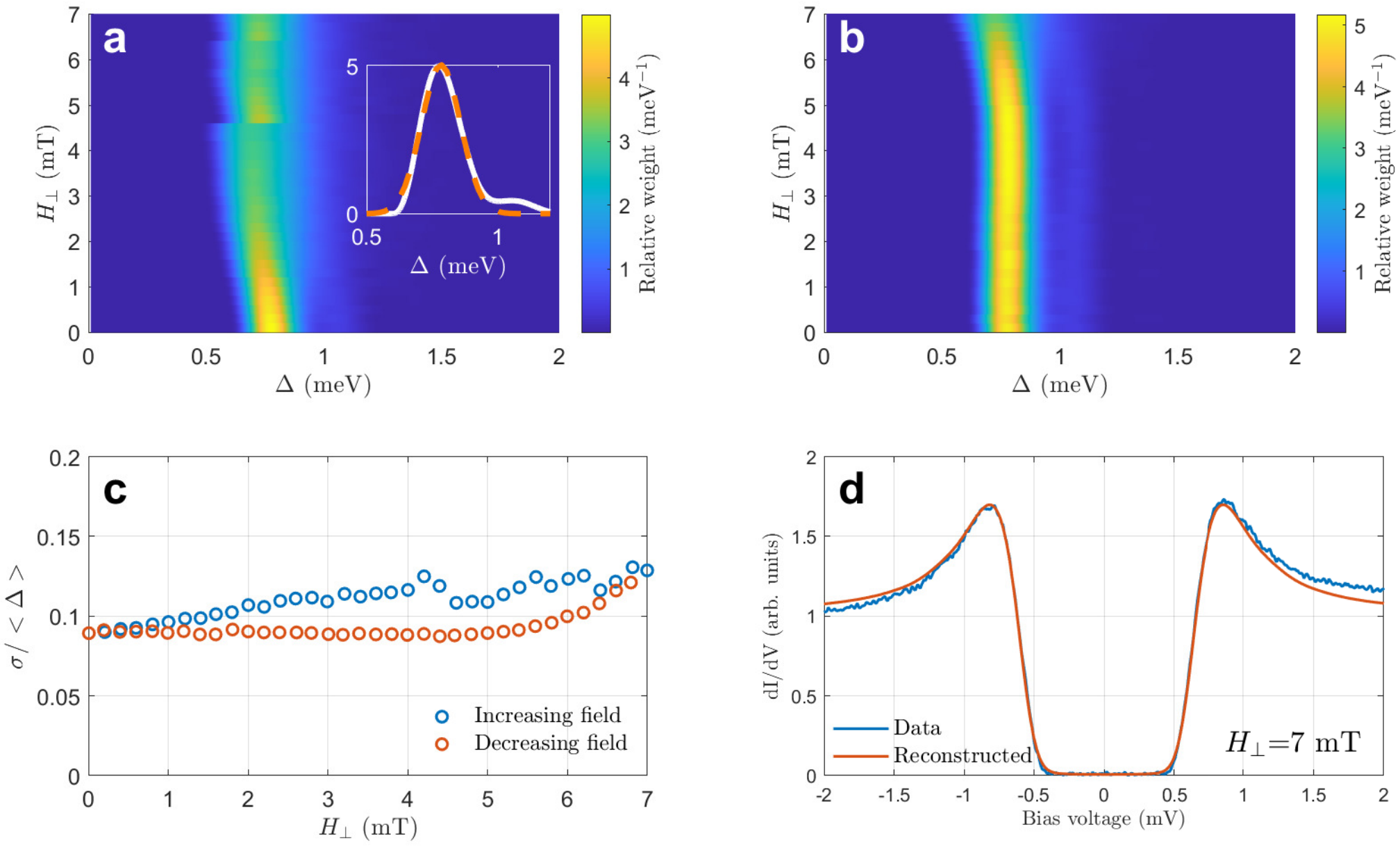

Figure S2: **Spectral decomposition of Junction 1.** The gap decomposition of Junction 1 in (a) increasing and (b) decreasing magnetic fields. Inset: the decomposition of the zero-field spectrum with overlaid Gaussian fit in dashed orange. (c) The width of the Gaussian fits normalized by their mean, showing a very small broadening of the distribution throughout the measurement. (d) The zero-field spectrum, overlaid with the fit reconstructed by the distribution, capturing the in-gap data well.

of the flake and thus resulting in a broader spread of the current magnitude. Another possibility is an accumulation of vortices closer to junction 3 than to the others, which creates a pinch point of the current along the edge, leading to the same effect. In panel d we verify that the different behavior is not due to a failure of the procedure, as it reproduces the spectra well both at zero and at maximal fields.

5 Current maps of single vortices at zero field

In Fig. S4 we display four current maps calculated using SuperScreen[2] at zero applied field with a single penetrating vortex, for the geometry of the 15-layer flake discussed in the main text. The position of the vortex, and consequently its distances from the four electrodes, is different in each panel. When the vortex is located at the very tip of the leftmost electrode (panel a), a considerable current density is induced under this electrode, on the order of the Meissner current at 2 mT. As the vortex grows more distant (panels b-d) the induced current density decreases. However, for the intermediate distances shown in panels b and c, the average current in the electrodes is a non-negligible fraction of the Meissner current we detect in our measurements. As a result, we conclude that a moderately close vortex induces a current that is well within the detection capabilities of our measurements.

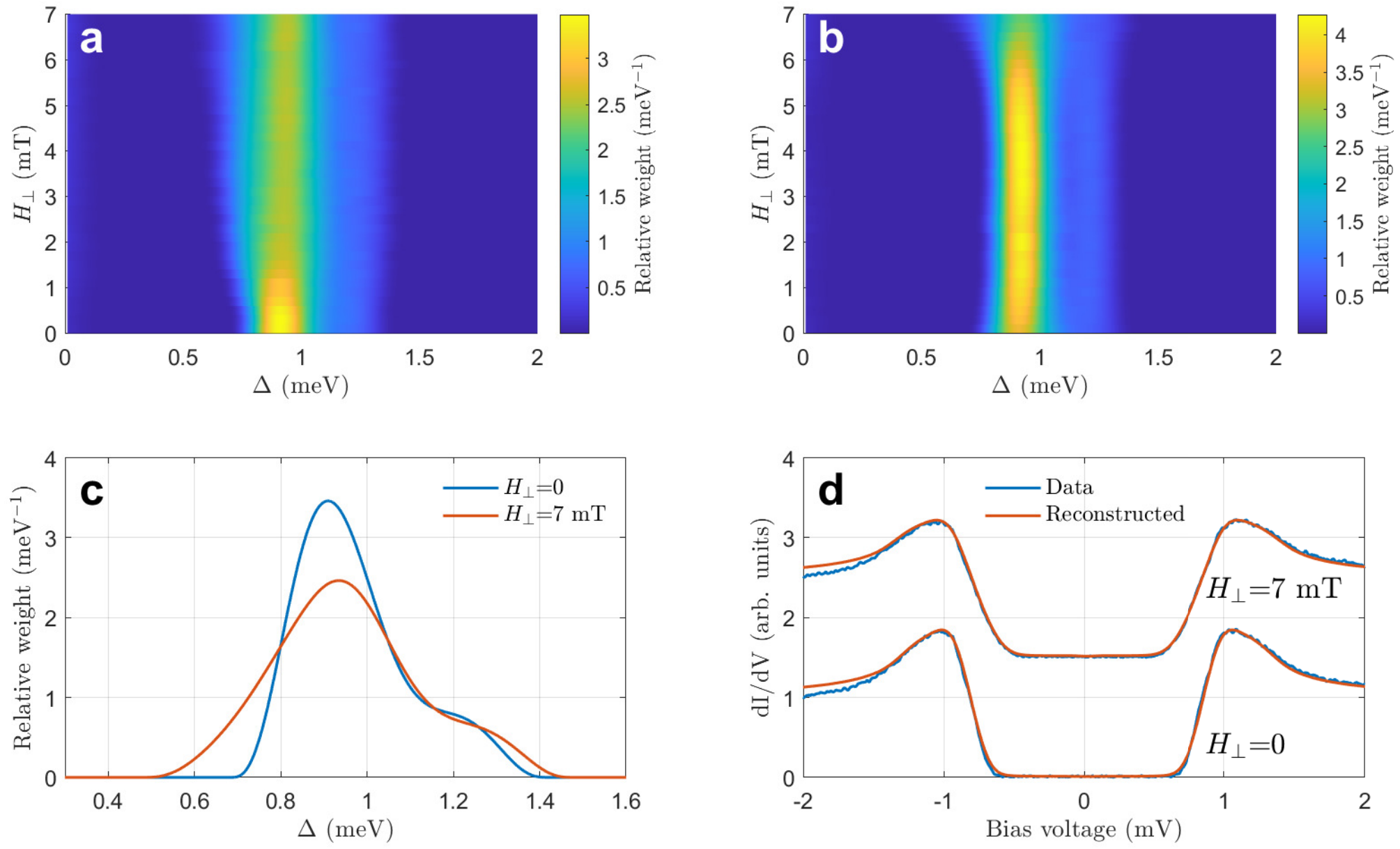

Figure S3: **Spectral decomposition of Junction 3.** The gap decomposition of Junction 3 in (a) increasing and (b) decreasing magnetic fields. Note the absence of horizontal shifting of the lobe at all fields. (c) Line cuts taken from panel a at zero field (blue) and at $H_\perp$ =7 mT (red), showing noticeable broadening of the distribution and indicating the redistribution of current under the junction. (d) The zero-field and $H_\perp$ =7 mT spectra, overlaid with the fits reconstructed by the distribution.

6 The effect of a single vortex at finite field

In figure S5 we display the current density calculated using SuperScreen[2] (panel a) for the case of a single vortex penetrating at 1 mT, at a distance of 280 nm from Junction 1 in the 15-layer flake. The calculated current density of Junction 1 as a result of a vortex penetrating closer, at a distance of 140 nm, is shown in green. The current map in the vicinity of the electrode before and after the penetration of a vortex 280 nm from Junction 1 is displayed in panels b and c, respectively. We conclude that the various small jumps in the measured V_{exit} are well explained by nearby vortex penetration.

7 The effect of multiple vortices at finite field

Figure S6a shows the predicted effect of a multiple-vortex penetration event on the current density under the electrodes in our flake, calculated using SuperScreen[2]. Panels b and c show the current density map at applied fields before and after the penetration of nine vortices, respectively, several μm away from the junctions. The colors in panel a correspond to the colors of the electrodes in b and c. A jump in the current density is apparent in the data, coinciding with the vortex penetration event, and it is clearly detectable in all but the farthermost electrode. This simulation strongly indicates that such multiple-penetration events are a likely explanation for several sharp jumps in our data.

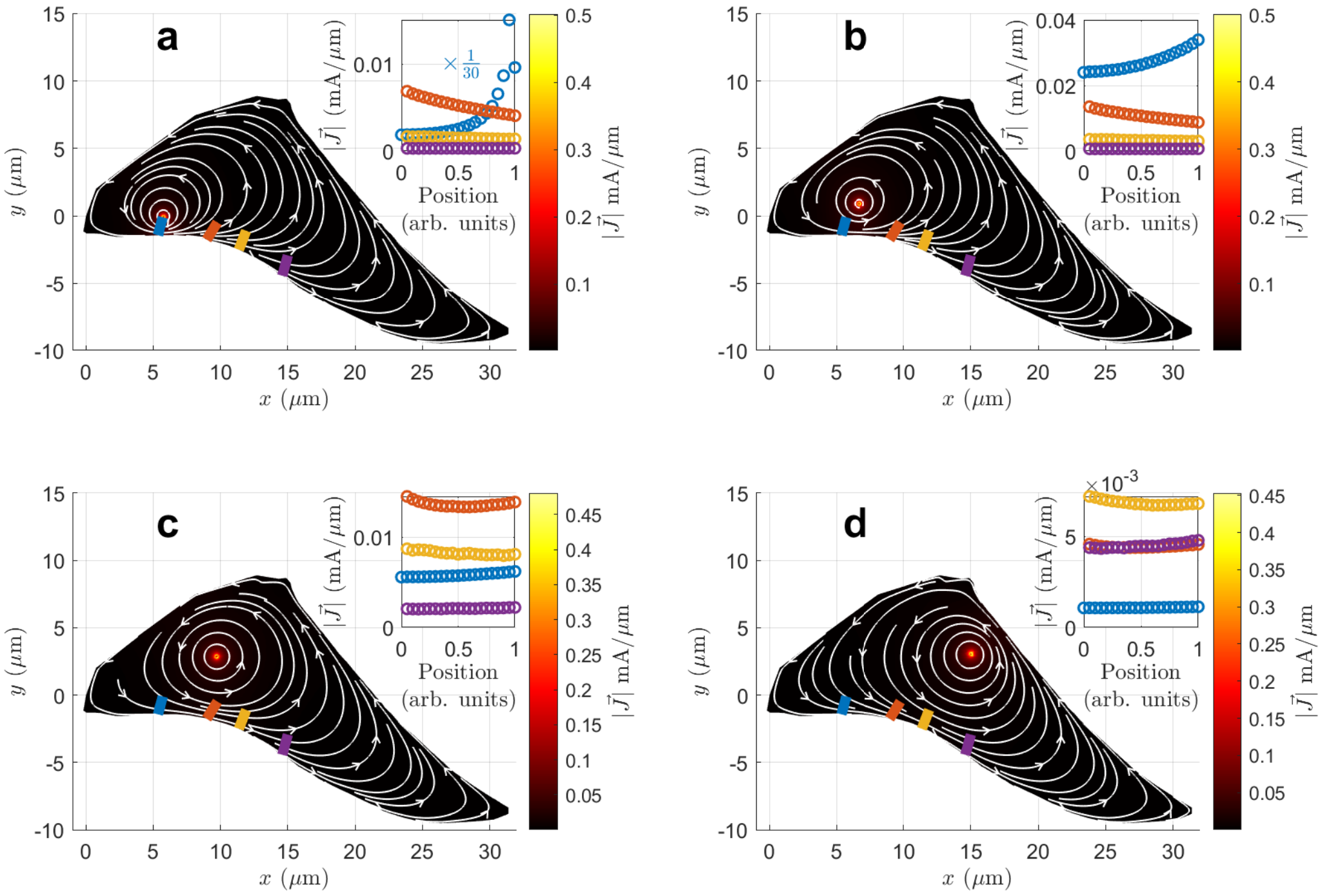

Figure S4: **Single vortex maps.** Current density maps, calculated using SuperScreen[2] at zero field with a single vortex penetrating at four different locations in panels a-d. Insets: current density profiles taken along the approximate positions of our electrodes. Horizontal axis normalized by the length of the junctions, $\approx 1\mu$m.

References

(1) Simon, S.; Klang, M.; Millo, O.; Steinberg, H. *Physical Review Letters* **2026**, *137*, 016001.

(2) Bishop-Van Horn, L.; Moler, K. A. *Computer Physics Communications* **2022**, *280*, 108464.

(3) Tinkham, M., *Introduction to superconductivity*; Courier Corporation: 2004.

(4) Khestanova, E.; Birkbeck, J.; Zhu, M.; Cao, Y.; Yu, G. L.; Ghazaryan, D.; Yin, J.; Berger, H.; Forró, L.; Taniguchi, T.; Watanabe, K.; Gorbachev, R. V.; Mishchenko, A.; Geim, A. K.; Grigorieva, I. V. *Nano Letters* **2018**, *18*, 2623–2629.

(5) Tikhonov, A. N. *Sov Dok* **1963**, *4*, 1035–1038.

(6) Tikhonov, A.; Arsenin, V., *Solutions of Ill-posed Problems*; Halsted Press book; Winston: 1977.

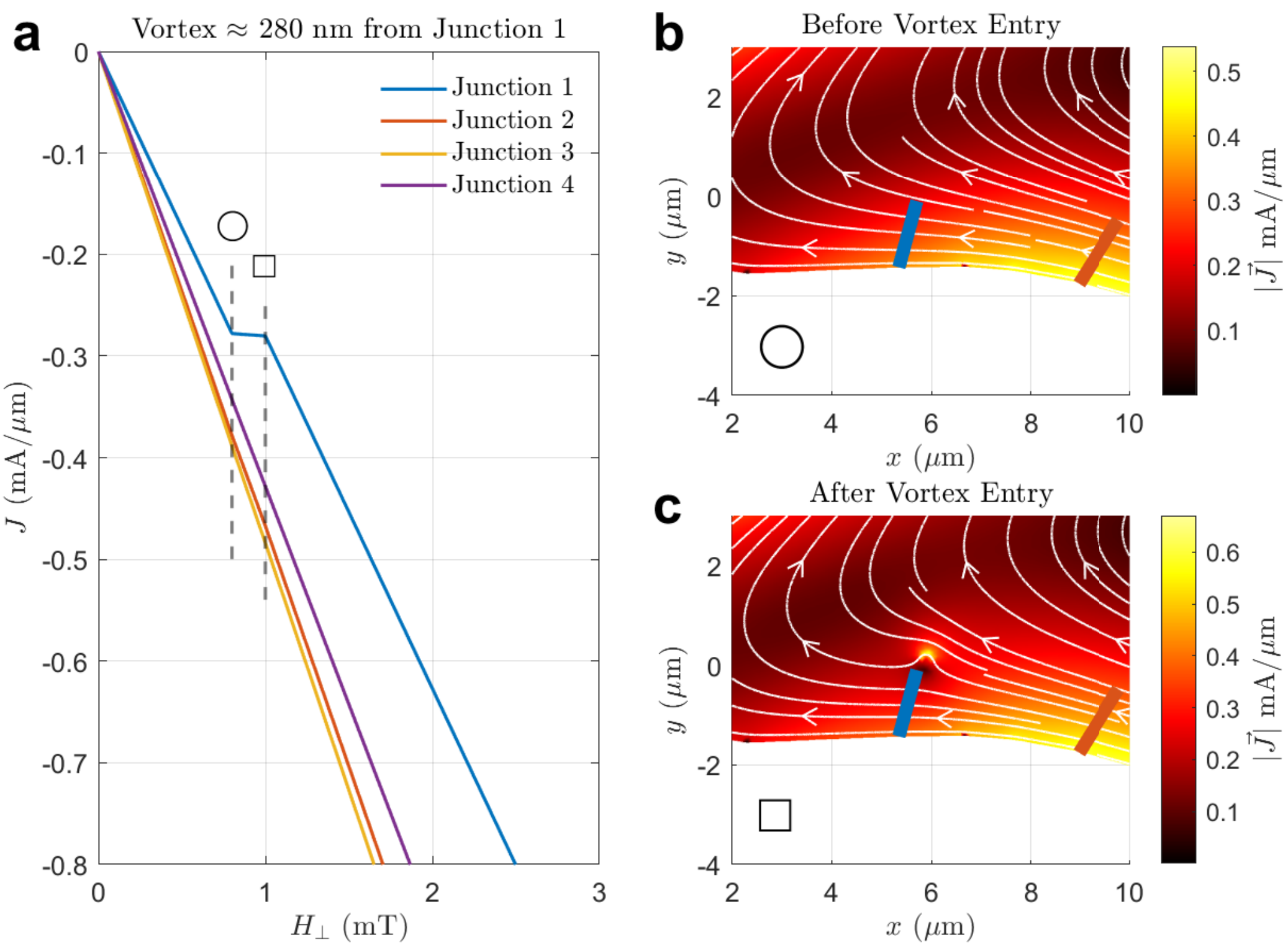

Figure S5: **Single vortex in finite field.** (a) Calculated current density of junctions 1-4 in the 15-layer flake, with a single vortex penetrating at a distance of 280 nm at $H_\perp$ =1 mT. (b) The calculated current map at $H_\perp$ =0.8 mT, before the vortex penetration. (c) The calculated current map at $H_\perp$ =1 mT, after the penetration of a vortex 280nm away from Junction 1. The circle and square icons in all panels mark the fields just before and after vortex penetration, respectively.

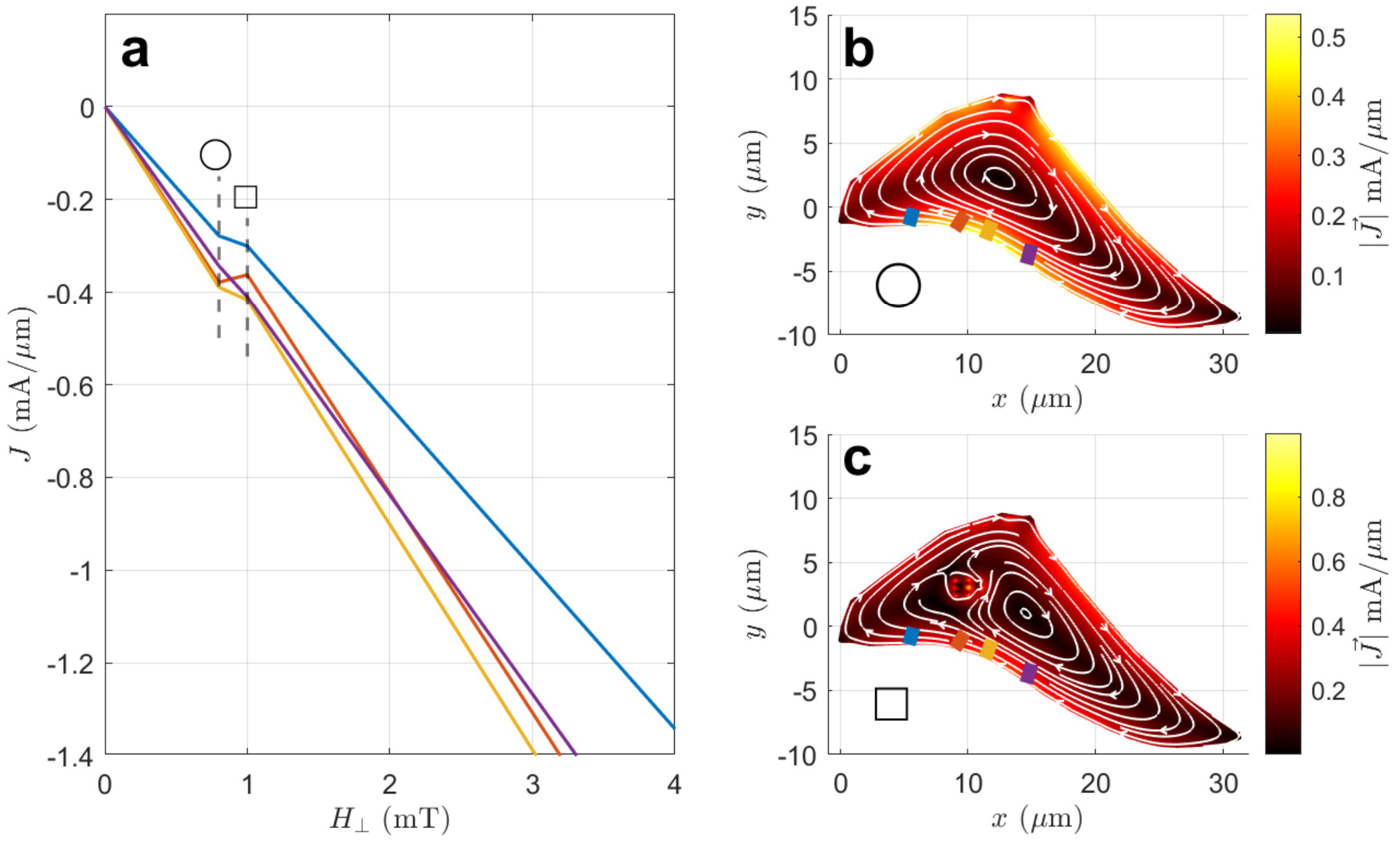

Figure S6: **The effect of the penetration of several vortices on the current density.** (a) Calculated current density profiles under each electrode, in colors corresponding to panels b and c. Nine vortices penetrate at a field of 1 mT, indicated by a square, leading to a noticeable jump in the data that is weaker in farther electrodes. The calculated field just below the jump is indicated by a circle. (b) The current density map calculated at the field just before the penetration event. (c) The current density map calculated at the field just after the penetration event, with the vortices clearly visible and the current field noticeably distorted.
